# Clinical, Biochemical, and Genetic Heterogeneity in Glutaric Aciduria Type II Patients

**DOI:** 10.3390/genes12091334

**Published:** 2021-08-27

**Authors:** Amanat Ali, Fatmah Saeed Ali Almesmari, Nahid Al Dhahouri, Arwa Mohammad Saleh Ali, Mohammed Ahmed Ali Mohamed Ahmed Aldhanhani, Ranjit Vijayan, Amal Al Tenaiji, Aisha Al Shamsi, Jozef Hertecant, Fatma Al Jasmi

**Affiliations:** 1 Department of Genetics and Genomics, College of Medicine and Health Sciences, United Arab Emirates University, Al Ain P.O. Box 15551, United Arab Emirates; amanat.a@uaeu.ac.ae (A.A.); 201505025@uaeu.ac.ae (F.S.A.A.); 202090177@uaeu.ac.ae (N.A.D.); 201602633@uaeu.ac.ae (A.M.S.A.); 201404437@uaeu.ac.ae (M.A.A.M.A.A.); 2 Department of Biology, College of Science, United Arab Emirates University, Al Ain P.O. Box 15551, United Arab Emirates; ranjit.v@uaeu.ac.ae; 3 Department of Pediatrics, Sheikh Khalifa Medical City, Abu Dhabi P.O. Box 51900, United Arab Emirates; aaltenaiji@seha.ae; 4 Department of Pediatrics, Tawam Hospital, Al Ain P.O. Box 15551, United Arab Emirates; aishamsi@seha.ae (A.A.S.); jhertecant@seha.ae (J.H.)

**Keywords:** electron transfer flavoprotein dehydrogenase, glutaric aciduria type II, metabolic acidosis, recurrent vomiting

## Abstract

The variants of electron transfer flavoprotein (*ETFA*, *ETFB*) and ETF dehydrogenase (*ETFDH*) are the leading cause of glutaric aciduria type II (GA-II). In this study, we identified 13 patients harboring six variants of two genes associated with GA-II. Out of the six variants, four were missense, and two were frameshift mutations. A missense variant (*ETFDH*:p.Gln269His) was observed in a homozygous state in nine patients. Among nine patients, three had experienced metabolic crises with recurrent vomiting, abdominal pain, and nausea. In one patient with persistent metabolic acidosis, hypoglycemia, and a high anion gap, the *ETFDH*:p.Gly472Arg, and *ETFB*:p.Pro94Thrfs*8 variants were identified in a homozygous, and heterozygous state, respectively. A missense variant *ETFDH*:p.Ser442Leu was detected in a homozygous state in one patient with metabolic acidosis, hypoglycemia, hyperammonemia and liver dysfunction. The *ETFDH*:p.Arg41Leu, and *ETFB*:p.Ile346Phefs*19 variants were observed in a homozygous state in one patient each. Both these variants have not been reported so far. In silico approaches were used to evaluate the pathogenicity and structural changes linked with these six variants. Overall, the results indicate the importance of a newborn screening program and genetic investigations for patients with GA-II. Moreover, careful interpretation and correlation of variants of uncertain significance with clinical and biochemical findings are needed to confirm the pathogenicity of such variants.

## 1. Introduction

Inborn errors of metabolism (IEM) constitute a rare heterogeneous set of genetic diseases with a wide range of overlapping or non-specific clinical phenotypes [1]. The complexity of metabolic pathways is increased by several proteins that play regulatory and enzymatic functions. IEM generally appears due to genetic mutations that disrupt the normal functioning of important proteins involved in cellular metabolism [2]. Glutaric aciduria type II (GA-II), also known as multiple acyl-CoA dehydrogenase deficiency (MADD) is an inborn error of fatty acid, choline, and amino acid metabolism that primarily affects fat and protein metabolism [3]. It is inherited in an autosomal recessive manner. Mostly, GA-II is caused by a defect in the electron transfer flavoprotein subunits (ETFA and ETFB), and the electron transfer flavoprotein dehydrogenase (ETFDH) [4].

Clinical phenotypes of GA-II are mostly classified into three types: two neonatal-onset forms (type I and type II) and a late-onset form (type III). GA II has a wide range of clinical manifestations, ranging from severe neonatal to moderate late-onset [5]. The neonatal form is typically more lethal, and patients often die during this time. However, the age of onset and clinical phenotypes are highly heterogeneous in the mild and/or late-onset forms, with irregular metabolic decompensation, and often make clinical diagnosis challenging [6]. The majority of existing clinical diagnoses are identified based on abnormal biochemical findings: characteristic urinary profiles of organic acids (including acylglycines), usually obtained using gas chromatography-mass spectrometry, and/or elevated plasma levels of short-, medium-, and long chain acylcarnitines, detected using tandem mass spectrometry methods [7]. A rise in acylcarnitine levels may occur during metabolic decompensation in patients with a mild form of the disease.

Several dehydrogenation reactions involved in MADD are disrupted due to impaired electron transfer of different flavoprotein dehydrogenases to the mitochondrial respiratory chain. Most flavoprotein dehydrogenase enzymes use flavin adenine dinucleotide (FAD) as the redox prosthetic group. Dehydrogenase enzymes are primarily involved in the oxidation of fatty acids and amino acids. Briefly, electrons are transferred from the prosthetic group of FAD to ubiquinone in the respiratory chain due to the involvement of ETF and ETF:ubiquinone oxidoreductase (ETF:QO). ETF is a heterodimer of α and ß-subunits that is located in the mitochondrial matrix and is composed of one FAD prosthetic group and one adenosine 5′-monophosphate (AMP) [8]. ETF:QO is a monomer encoded by the *ETFDH* gene, located in the inner mitochondrial membrane, and comprises three closely connected functional regions, a 4Fe4S cluster, a FAD binding domain, as well as a UQ binding domain [9]. The ETF and ETF:QO proteins are cytosolically imported like other mitochondrial enzymes and the binding process of FAD occurs inside the mitochondria. Protein’s fold into their normal conformation in the mitochondria [10].

Multiple studies have demonstrated that patients with MADD generally have mutations in *ETFA, ETFB* or *ETFDH* genes [11,12,13]. According to the Human Gene Mutation Database (HGMD), over 190 different variants of *ETFDH* have been reported so far and most of them are associated with the late-onset form of GA-II [14,15,16]. The majority of patients presented with the late-onset form are responsive to riboflavin therapy, but the molecular mechanism remains elusive. The genotype and phenotype correlation in GA-II patients with *ETFDH* mutation has been proposed in the past. A large-scale study on patients with the late-onset mild form however failed to establish such a correlation [16].

In this study, we report the clinical, biochemical, and genetic heterogeneity in a cohort of GA-II patients and evaluate the pathogenicity of variants present in genes linked with GA-II. The main objective of this study is to re-classify the variants of unknown significance in patients with GA-II and provide clinical, biochemical, and computational data.

## 2. Materials and Methods

### 2.1. Ethical Consideration and Data Collection

Ethical approval for this retrospective data collection study was obtained from the Tawam Human Research Ethics Committee (THREC-584), however consent taking was exempted. Patients were recruited through a metabolic clinic at Tawam Hospital, Abu Dhabi, United Arab Emirates. Clinical and biochemical data collection was performed as per allowed national guidelines. GA-II screening was made based on the presence of elevated levels of C4 and C5 +/− and other acylcarnitines as per the American College of Medical Genetics (ACMG) algorithm detected during a newborn screening (NBS) program using tandem mass spectrometry [17]. Patients who had a positive NBS for GA-II and/or clinical features suggestive of GA-II were recruited and further confirmatory work-up was performed as per the recommendations of ACMG at Tawam Hospital. Genetic investigations for affected patients with clinical presentation of MADD were requested and diagnostic gene panel sequencing or whole exome sequencing (WES) was performed by Centogene AG (Rostock, Germany). A review of the variants associated with GA-II (identified by genetic analysis between February 2009 and December 2020) was performed between 1 February 2021 and 12 April 2021).

### 2.2. Computational Analysis of Variants

Twelve in silico tools were used to determine the pathogenicity of identified missense variants. The tools used were SIFT (Sorting Intolerant from Tolerant) [18], Polyphen-2 (Polymorphism Phenotyping v2) [19], LRT (likelihood ratio test) [20], Mutation Taster [21], Mutation Assessor [22], FATHMM (functional analysis through hidden markov models) [23], PROVEAN (Protein Variation Effect Analyzer) [24], MetaSVM [25], MetaLR [26], REVEL (Rare Exome Variant Ensemble Learner) [27], CADD (Combined Annotation-Dependent Depletion) [28], DANN (Deep Neural Network) [29], and Condel (Consensus Deleterious) [30]. The putative post-translation modification (PTM) sites in the human ETF-QO and ETFB proteins were identified using GPS-MSP 1.0 [31], iPTMnet [32], and PhosphoSitePlus [33]. Mutpred2 was used to analyze the deleterious effect of amino acid substitutions and their impact on the molecular mechanisms [34]. Additionally, InterPro was used to determine the location of missense variants on the conserved domains of ETF-QO [35].

### 2.3. Evaluation of the Effect of Missense Variants on the Protein Stability

To predict the effect of single point mutations on protein stability, the I-Mutant tool was used [36]. Input data of missense variants of *ETFDH* were submitted in FASTA format. Moreover, HOPE (Have (y)Our Protein Explained), a web-based server was also used to analyze the structural effects of single point mutations in a protein sequence [37]. The amino acid sequence of ETF-QO retrieved from UniProt (Accession No. Q16134) and four missense variants produced manually were used as input. The effect of missense variant on the secondary structure of ETF-QO was also evaluated using PSIPRED [38]. Additionally, the Missense 3D tool was used to measure the structural changes caused by point mutations.

### 2.4. Evaluation of Protein Evolutionary Conservation

The ConSurf server was used to determine the evolutionary conservation of each amino acid in a macromolecule [39]. The wild type amino acid sequence was submitted as the input. Multiple sequence alignment (MSA) of human ETF-QO with 20 different organisms was also performed to determine the conservation of substituted residues at specific positions and calculate Jensen-Shannon Divergence (JSD) scores [40]. ETF-QO protein sequences from human, chimpanzee, hamster, mouse, rat, rabbit, cat, fox, horse, pig, bat, goat, bovine, elephant, panda, chicken, alligator, whale, shark, and fruit fly were retrieved from UniProt, and MSA was performed using ClustalW [41]. Aligned sequences were exported in FASTA format to measure JSD scores.

### 2.5. Evaluation of the Effects of Missense Variants Using Three-Dimensional Protein Modeling

Modeller10.1 was used to generate homology models of ETF-QO and mutant proteins [42,43]. The protein sequence of human mitochondrial ETF-QO was obtained from UniProt (Accession number: Q16134). The retrieved 3D crystal structure of porcine ETF-QO (PDB: 2GMH) from Protein Data Bank (PDB) was used as a template for producing homology models of wild type and mutant human ETF-QO structures. PyMOL was used to evaluate and visualize the generated models [44].

## 3. Results

### 3.1. Clinical, Biochemical, and Genetic Characteristics of the Studied Patients

Patients who had a positive newborn screening (NBS) for GA-II and/or clinical features suggestive of GA-II along with a diagnostic mutational analysis report were included in this retrospective study. A total of 13 patients fulfilled this criterion and were further investigated.

**Figure 1:**
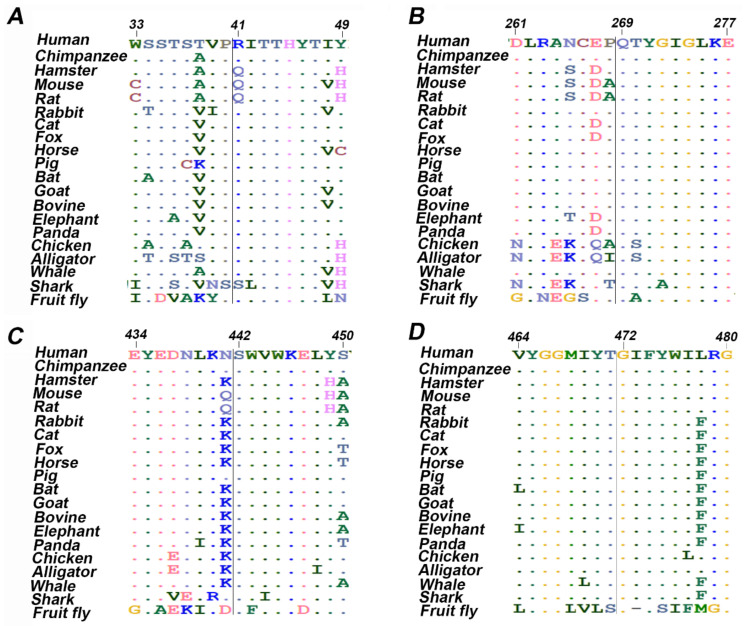
Multiple sequence alignment of twenty amino acids centered on the missense mutation obtained from different organisms. (**A**) ETF-QO, R41L; (**B**) ETF-QO, Q269H; (**C**) ETF-QO, S442L; (**D**) ETF-QO, G472R.

**Figure 2:**
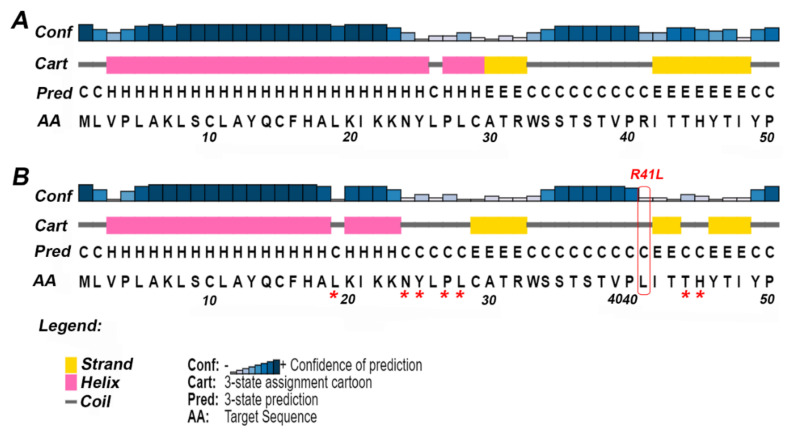
The secondary structure of ETF-QO predicted by PSIPRED. For clarity, only the effect of the missense variant Arg41Leu has been shown. The predicted full-length sequence of ETF-QO is shown in Supplementary Figure S2. (**A**) Secondary structure of wild type ETF-QO; (**B**) secondary structure of ETF-QO with R41L variant. The square box shown in red represents the location of mutation and the asterisk (*) shows the position of change in secondary structure in mutant ETF-QO.

**Figure 3:**
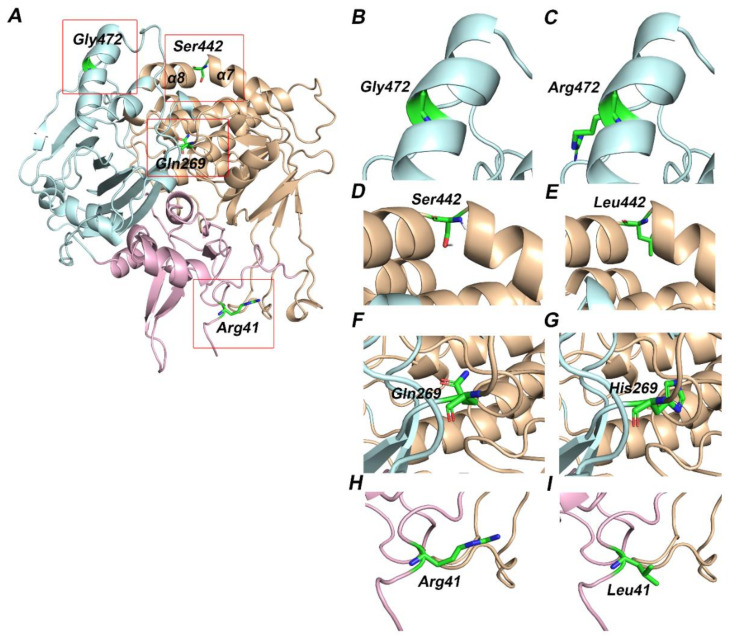
Three-dimensional structure of ETF-QO protein. The functional domains of ETF-QO protein—FAD (orange), UQ (cyan), and 4FE4S cluster (pink) are shown in cartoon representation and amino acids are shown in stick representation. The red boxed regions in A are magnified in the successive images. (**A**) Modeled structure of ETF-QO; (**B**) wild type Gly472; (**C**) mutant Arg472; (**D**) wild type Ser442; (**E**) mutant Leu442; (**F**) wild type Gln269; (**G**) mutant His269; (**H**) wild type Arg41; (**I**) mutant Leu41.

Case 1 is asymptomatic, and he did not experience any episode of metabolic crises (Table 1). He was identified through NBS. The results of plasma acylcarnitine obtained during NBS confirmatory work-up were suggestive of GA-II (Table 2). He was managed with riboflavin. The diagnostic mutational analysis was ordered as part of positive NBS confirmatory workup for GA-II. Results identified a novel homozygous missense variant *ETFDH*:p.Arg41Leu. This variant has not been reported in any database to the best of our knowledge. It is classified as VUS according to the recommendations of ACMG. Homozygosity was confirmed by parental testing. *ETFDH*:p.Arg41Leu has conflicting pathogenicity predictions, primarily due to low scores of Polyphen-2, Mutation Assessor and MetaSVM (Table 3). Arg41 is not conserved; it is replaced by glutamine in multiple species (Table 3 and Figure 1A). Leu41 variant also altered the secondary structure of the surrounding residues (Figure 2). It is located in the 4FE4S cluster (Figure 3H). The change to hydrophobic Leu41 is physiochemically significant. However, its location and lack of intramolecular interactions may not affect the protein. This could be the likely cause of the patient’s abnormal acylcarnitine. The clinical significance of this variant is not known.

*ETFDH*:p.Gln269His was found in nine patients (cases 2–10). Among nine patients, three (cases 2–4) have experienced metabolic crises with recurrent vomiting, abdominal pain, and nausea. However, the onset of metabolic crises was different in all three cases (Table 1). Case 2 had metabolic crises at the age of 26 years. He presented with recurrent vomiting, epigastric pain, and nausea. His vomiting was persistent, and reports of biochemical results indicated severe metabolic acidosis. Intravenous fluids were administered but there was no improvement. The patient developed a coma over time, and ammonia levels were also found to be significantly elevated. He developed encephalopathy, which required several days of dialysis to reduce his ammonia levels. Additionally, pulmonary embolism, ischemia and elevated concentrations of liver enzymes were observed (Table 2). WES results indicated the presence of the *ETFDH*:p.Gln269His variant. Case 3 was born before the implementation of expanded NBS. She presented with acute metabolic acidosis, recurrent vomiting, and abdominal pain at the age of 20 months. She also experienced hypoglycemia and seizure attacks. Urine organic acid profile during metabolic crisis was consistent with GA-II and genetic testing identified the *ETFDH*:p.Gln269His variant at the age of 7 years. Case 4 is a sibling of case 3 and she experienced metabolic crises when she was 15 years old. However, the presence of the *ETFDH*:Gln269His variant was confirmed during a family screening at the age of 5 years. She had a history of recurrent vomiting, epigastric pain, and metabolic acidosis (Table 1). Her non-compliance with the medication regimen could be the reason for her recurrent metabolic acidosis. Her GA-II related complications were managed with carnitine and riboflavin. Homozygosity of the *ETFDH*:p.Gln269His variant was confirmed in all cases by parental testing. In silico tools consistently predicted this variant as pathogenic. Gln269 is a highly conserved residue (Table 3 and Figure 1B). It is located at the boundary of FAD and UQ binding domains (Figure 3F). Histidine has different physicochemical properties, and the His269 variant is likely to affect the structure and intermolecular interactions in ETFO-QO protein. Its ClinVar ACMG classification is VUS. Here, the clinical information and biochemical findings of these patients confirm the pathogenicity of this variant.

The remaining six patients (cases 5–10) of the *ETFDH*:p.Gln269His variant were either asymptomatic or had a mild form of GA-II with no history of metabolic crises (Table 1 and Table 2). Cases 5 and 6 are siblings of case 3 and 4. Both are confirmed cases of mild GA-II, and the *ETFDH*:p.Gln269His variant was detected as part of family screening. Case 5 has asthma and a mild form of GA-II. Her biochemical results indicated abnormal acylcarnitine and carnitine profiles suggestive of GA-II (Table 2). Case 6 has a history of occasional vomiting episodes, carnitine deficiency, and cardiac murmur. Her urine organic acid results were found normal, while the acylcarnitine profile was not performed (Table 1 and Table 2). Case 7 has a history of recurrent viral infections and vomiting since birth. Her NBS report for GA-II was positive. Results of acylcarnitine profiles were suggestive of GA-II. Case 8 and 9, are asymptomatic patients with GA-II with abnormal acylcarnitine and carnitine profiles (Table 1 and Table 2). Case 10 has myopathy, hypoglycemia, recurrent vomiting, and nausea. Biochemical results indicated abnormal levels of acylcarnitine, indicative of GA-II. Genetic testing was performed as part of positive NBS confirmatory workup for GA-II and the results identified the *ETFDH*:p.Gln269His variant in case 7, 8, and 9. GA-II related complications of these patients were managed with carnitine and/or riboflavin.

Case 11 was born before the implementation of expanded NBS. She presented at 3 months of age with poor feeding, vomiting and hypotonia. Subsequently, she developed severe metabolic acidosis with normal anion gap, hypoglycemia, hyperammonemia and liver dysfunction (Table 1 and Table 2). Her urine organic acid profile was consistent with GA-II. High doses of riboflavin and carnitine were ordered to manage her GA-II related complications. However, she did not respond to the treatment regimen and died at the age of 4 months after developing acute respiratory distress, renal failure, sepsis, and disseminated intravascular coagulation (DIC). During the management, gene sequencing was ordered as part of confirmatory workup for GA-II. Sequencing results identified a homozygous missense variant *ETFDH*:p.S442L. In silico tools consistently predicted this variant as pathogenic (Table 3). Ser442 is a highly conserved residue (Table 1 and Figure 1C). It is located at FAD domain (Figure 3D). It is replaced by a hydrophobic residue, Leu442. It is classified as VUS according to the recommendations of ACMG. Here, the clinical information and biochemical findings confirm the pathogenicity of this variant.

Case 12 was admitted to the hospital on the second day after birth with a strong suspicion of metabolic acidosis. NBS was not performed. She had persistent metabolic acidosis, grunting, and hypoglycemia since birth. Arterial blood gas (ABG) test results indicated severe metabolic acidosis with low bicarbonate levels and high anion gap (Table 1 and Table 2). High levels of creatinine, ammonia, and urea were also found. She often reported hypotonic, awake but unresponsive, and had abnormal breathing patterns at rest. Results of urine organic acids and acylcarnitine profile were found elevated. High doses of IV fluids, sodium benzoate, phenylbutyrate, acetate, 3-hydroxybutyrate, and multivitamins (riboflavin, glycine, and carnitine) were used to manage her acidosis related complications. However, her metabolic acidosis, high anion gap, and hyperammonemia remained persistent. Despite receiving maximum doses, her condition continuously deteriorated, and she died on the fifth day of her hospitalization. WES was ordered to determine the underlying genetic diagnosis of metabolic acidosis, high anion gap, and hyperammonia. Results identified the *ETFDH*:p.G472R variant in a homozygous state. In silico tools predicted this variant as pathogenic (Table 3). Gly472 is a highly conserved residue (Table 3 and Figure 1D). It is replaced by Arg472, which is a basic residue and has high side chain flexibility. It is classified as likely pathogenic based on ClinVar ACMG classification. Additionally, a frameshift mutation *ETFB*:p.Pro94Thrfs*8 was also found in this patient in a heterozygous state. Mutation Taster predicted this variant as benign. Its clinical significance is unknown, ClinVar classifies it as VUS. Here, clinical, biochemical, and in silico evaluations indicate the pathogenicity of *ETFDH*:p.G472R. It is likely pathogenic, in agreement with its ClinVar ACMG classification.

Case 13 was born before the implementation of expanded NBS. She has a long history of recurrent vomiting and symptomatic hypoglycemic episodes associated with seizures. However, she did not experience any episodes of metabolic crises. Urine organic acids concentrations and acylcarnitine profile were found elevated (Table 1 and Table 2). Her GA-II related complications were managed with carnitine and riboflavin. WES was ordered at the age of four years as part of workup for recurrent hypoglycemia and seizure. Sequencing results identified a novel frameshift mutation *ETFB*:p.Ile346Phefs*19 in a homozygous state. This variant has not been reported in any database to the best of our knowledge. Mutation Taster predicted this variant as benign. However, this mutation was predicted to induce alteration in the splice site region. *ETFB*:p.Ile346Phefs created a gain of donor splice site at gDNA position 21203. Its clinical significance is unknown. Here, the clinical findings indicate pathogenicity.

### 3.2. In Silico Analysis of Identified Variant Pathogenicity

In this study, six variants were found in two genes (*ETFDH* and *ETFB*) associated with GA-II, recurrent vomiting, and hypoglycemia. Among six variants, four are missense and two frameshifts. Missense variants were observed in *ETFDH* gene and were predicted as pathogenic, while frameshift variants were found in *ETFB* gene and were predicted benign (Table 3). The effect of variants on putative PTM sites was also evaluated. However, no variant was observed to be present at sites identified to be post translationally modified. Among the two frameshift mutations, *ETFB*:c.278dup (p.Pro94Thrfs*8) was found in one patient (Table 1). Mutation Taster predicted this as benign. This variant is also reported in ClinVar with seemingly polymorphic frequencies in some population. Therefore, this mutation is probably benign. *ETFB:*c.1035del (p.Ile346Phefs*19), a novel variant was detected in one patient (Table 1). Since this variant is positioned at the very end of the coding sequence and no similar variants are known in the literature as pathogenic. Therefore, its clinical significance is not known.

Furthermore, the effect of four missense variants on protein function and structure was analyzed computationally. FannsDB Condel was performed to evaluate the functional annotation of these missense variants. Results predicted these variants as pathogenic (Table 3). MutPred2 was also used to predict the effect of missense variants and their likelihood to alter the molecular mechanisms and damaging effect on protein (Supplementary Table S1). The computed g scores of all variants were greater than the threshold (0.5). This suggests that these variants are damaging to ETF-QO protein structure. Moreover, all missense variants were predicted to undergo molecular changes.

### 3.3. Evolutionary Conservation Analysis

ConSurf was performed to detemine the conservation of ETF-QO amino acids. The position of the variant in the wild type sequence is shown with red outlined boxes (Supplementary Figure S1). Arg41 and Gly472 were predicted to be solvent exposed and buried residues, respectively, with variable prediction of conservation. However, Glu269, and Ser442 were predicted to be highly conserved and solvent exposed residues. Additionally, MSA was carried out to validate the conservation of missense variants predicted by ConSurf using ClustalW (Figure 1). For this, amino acid sequences of human and different organisms were retrieved and aligned (Supplementary Table S3). JSD scores of each variant were also computed (Table 1). Results indicated that Arg41, Glu269, Ser442, and Gly472 amino acids are conserved. Therefore, it would be inferred that any substitution at these positions could likely produce damaging effect on the protein structure and function.

It has been reported that nearly 80% of the mutations involved in disease pathogenesis are located in the secondary structure of the protein [45]. Thus, careful evaluation of SNPs on the protein secondary structure is imperative to understand any changes in the tertiary structure of the protein. PSIPRED predicted the coiled structure of ETF-QO at position Arg41 and Glu269, while α helix at Gly472 (Figure 2). The variants Gln269His, Ser442Leu, and Gly472Arg did not produce any change in the secondary structure conformation. However, Arg41Leu variant altered the secondary structure of the surrounding residues (Figure 2A,B). This substitution disturbed the helical and strand regions of the ETF-QO. The conformation of residues the Leu19, Asn24-Tyr25, and Pro27-Leu28 from helical to coiled and Thr44-His45 from strand to coiled was observed (Figure 2).

### 3.4. Evaluation of The Structural Stability and Effect of Missense Variants on ETF-QO Protein

I-mutant Suite was used to determine the protein stability. Results indicated that all variants decreased the ETF-QO stability compared to wild type. Among four missense variants, S442L and Q269H were found to produce the highest instability in ETF-QO with a Gibbs free energy change value (ΔΔG) of −3.0 and −0.87 kcal/mol, respectively (Table 4). Additionally, HOPE was performed to evaluate the impact of variants on protein domains and functions. HOPE predicted these variants as highly damaging to ETF-QO protein (Supplementary Table S2). The analysis of Arg41Leu indicated that the substitution of arginine into a smaller residue, leucine, is likely to break hydrogen bonds and salt bridges and could disturb correct protein folding. In the case of the Glu269His variant, the mutant is more hydrophobic and bigger in size. This could introduce bumps in the protein structure. HOPE indicated that the mutant residue of Ser442Leu variant is bigger in size and has more hydrophobicity. Ser442 was predicted to be located in an intramembrane region. Studies have shown that these regions often have a special role in the protein stability and function [46,47]. Results of Gly472Arg variant revealed that arginine is a charged and hydrophilic residue compared to wildtype. The substitution of glycine, the most flexible residue into arginine could disturb the protein flexibility at this position. The structural flexibility and rigidity of the protein is considered important for producing vital functions. The introduction of charged residue at this position could also lead to repulsion of ligands or other residues with the same charge (Supplementary Table S2). Missense3D analysis indicated that the Gln269His variant introduced a buried charged residue and produced a contraction of cavity volume by 63.72 Å3, while Gly41Leu, and Gly472Arg variants caused an expansion of cavity volume by 33.264 Å3 and 86.61 Å3, respectively.

Additionally, homology models of wild type and variants of human ETF:QO protein were generated using Modeller10.1. Human ETF:QO protein showed 95% sequence identity with the porcine ETF:QO. Results indicated that wild-type residues Arg41 and Ser442 are located in the 4FE4S cluster and the FAD domain, respectively, while Gln269 and Gly472 are present in the UQ binding domain of the ETF-QO protein (Figure 3). These binding domains are evolutionarily conserved. Previous studies have demonstrated that mutations in these domains could hinder the binding interactions of 4FE4S, FAD and UQ with ETF:QO [48]. Residue Gln269 is located at the junction of FAD and UQ binding domains. It is perceivable that this region could be destabilized by His269 variant due to its polar interaction with Arg263. Ser442 is located on a kink that links helices α7 and α8 (Figure 3F). Ser442 forms a hydrogen bond with the backbone carbonyl group of Leu439. The hydrophobic sidechain of the Ser442Leu variant is likely to disrupt this interaction (Figure 3D,E). Gly472 is a buried residue located on the α-helical region of the UQ domain (Figure 3B).

## 4. Discussion

This study reports the association of electron transfer flavoprotein mutations in a cohort of GA-II patients. Almost 60% (8/13) of the patients came from consanguineous families, and the rest is not known, but all variants were homozygous in nature, suggestive of consanguinity (Table 1). The diagnosis of the patients was made based on their clinical and biochemical features. Results indicate that GA-II with or without recurrent vomiting, encephalopathy, metabolic acidosis, and hypoglycemia is linked significantly with likely pathogenic variants such as *ETFDH*:p.Gln269His, *ETFDH*:p.Ser442Leu, *ETFDH*:p.Gly472Arg, and ETF:p.Ile346Phefs*19. *ETFDH*:p.Arg41Leu variant has conflicting predictions of pathogenicity. Thus, the careful evaluation of the clinical phenotypes associated with these variants is imperative. Moreover, this study also identified a family of four siblings who were confirmed to have the *ETFDH*:p.Gln269His variant in a homozygous state. 

Importantly, the diagnosis of GA-II in adult cases is complex, and eventually leads to a notable diagnostic delay. Early diagnosis and management are critical for patients with acute decompensations to recover and have a positive outcome. The awareness among physicians treating GA-II in adults is significantly low since inborn errors of metabolism are generally perceived as pediatric diseases. Additionally, forming a differential biochemical diagnosis for the late onset form of GA-II is also difficult in both symptomatic and asymptomatic patients [49]. The majority of affected individuals exhibit a classical pattern of organic acids and acylcarnitines in urine and blood, respectively, especially during metabolic crises. However, profiles of acylcarnitine and organic acids could be observed to be unremarkable during metabolically stable conditions [50,51,52,53,54]. This suggests that a single metabolic screening test cannot rule out MADD completely. Therefore, a differential diagnosis should be based on blood and urine examinations, and eventually be confirmed by molecular approaches. Here, we have identified nine cases of late onset GA-II with different clinical presentations (Table 1). Interestingly, one case was identified as late as at the age of 30 years after first experiencing the symptoms (Table 1). 

The characterization of mutations as pathogenic is expected to enhance the overall clinical plan for patients with GA-II. Studies have demonstrated the positive impacts of early diagnosis by NBS and timely therapeutic intervention on the neurological outcomes [55,56,57,58]. As the age at which symptoms first appear is a strong predictor of the severity of the GA-II associated complications. Therapeutic management of asymptomatic patients has prevented the onset of the symptoms [59,60,61]. This suggests the importance of NBS and inclusion of molecular approaches for the early diagnosis of patients with atypical presentations. However, further efforts are needed to minimize the incidences of delayed diagnosis and treatment.

Previous studies have demonstrated that phenotype in a majority of the patients cannot be accurately predicted from genotype [62,63]. This could be due to a small number of patients or novel variants. Here, the *ETFDH*:p.Q269H variant was found in 69% (9/13) of the patients with GA-II (Table 1). In silico tools consistently predicted this as pathogenic. Therefore, a convincing genotype-phenotype correlation likely exists for this variant. 

The homozygous or compound heterozygous variants of *ETFA*, *ETFB*, and *ETFDH* genes are primarily associated with GA-II (6). Variants of ETFA and ETFB are generally linked with the neonatal forms, whereas *ETFDH* variants are mostly associated with the late-onset forms of GA-II [64]. However, the clinical presentations differ significantly in the late-onset forms of GA-II. The clinical manifestations of the late-onset form of GA-II range from acute, sometimes life-threatening metabolic crises during childhood to asymptomatic adults. Additionally, these clinical phenotypes can differ significantly even within the same family [12,65]. Patients harboring *ETFDH* variants mostly experience GA-II related symptoms during the first two decades. Patients with late-onset forms of GA-II are generally presented with chronic, and myopathic symptoms. However, approximately one third of such patients also exhibited metabolic decompensation or acute symptoms. Such metabolic decompensations are more common in infants; however, serious metabolic crises have also been reported in adults [66,67,68,69]. Decompensations are generally associated with acidosis, increased activities of transaminases, hypoglycemia, and rhabdomyolysis with increased creatinine kinase activity, and ultimately hyperammonemia. Catabolic states, such as infections and febrile diseases, or a drop in energy supply, are typically the cause of these episodes. Other triggering factors such as low-energy diets, alcohol, weight loss, menstrual period and pregnancy can also play a role in adults [51,70,71].

Several studies have identified hotspots in *ETFDH* gene and their association with GA-II [16,72,73,74]. Interestingly, the majority of GA-II patients have been reported from Asian countries. This study reports four missense variants of *ETFDH* associated with either metabolic crises or a mild form of GA-II (Table 1). Different bioinformatics tools predicted these variants to be pathogenic (Table 3). Evolutionary conservation analysis of protein sequences is essential for determining the deleterious effect of mutations on the protein structure and function. Here, the results of MSA and ConSurf indicated higher conservation scores for highly deleterious variants (Figure 1 and Supplementary Figure S1). Additionally, these variants were situated in conserved regions of ETF-QO protein (Figure 3). Therefore, any substitution at these positions could produce a damaging effect on the structure and function of the protein. Protein stability is often considered critical for its functional and structural activity [75]. Moreover, any change in protein stability can lead to its degradation or misfolding. Here, the results of I-Mutant indicated that all missense variants decreased the stability of ETF-QO (Table 4).

In conclusion, we described the *ETFDH* and *ETFB* variants associated with different forms of GA-II. It is obvious that the late onset form of GA-II patients produced acute metabolic crises. However, further studies are required to compute the prevalence of these variants in the community. Additionally, genetic investigations should be considered in the workup of the GA-II patients, especially in adults with atypical presentations. Results highlighted the importance of including genetic testing for the differential diagnosis of GA-II with unresolved phenotypes. In silico analysis may also aid in discovering and designing novel pharmaceutical chaperones that could regulate the mutant proteins to fold and route correctly.

## Figures and Tables

**Table 1:** Clinical characteristics and genotypes of the studied patients.

Case I.D	Clinical Phenotype	Gender	Age at Onset	Age at Diagnosis	Parents	Variant	Zygosity	Variant Detection
1	Asymptomatic; no metabolic crises	Male	NA	2 months	First cousins	*ETFDH:*c.112G > T p.Arg41Leu; missense	Homozygous	During NBS confirmatory workup
2	Metabolic crises; encephalopathy; hyperammonemia and metabolic acidosis; recurrent vomiting	Male	26 years	26 years	Second cousins	*ETFDH*:c.807C > T, p.Gln269His; missense	Homozygous	Based on clinical presentation; NBS was not performed
3	Metabolic crises; cyclic vomiting; abdominal pain, nausea	Female	20 months	7 years	First cousins	*ETFDH*:c.807C > T, p.Gln269His; missense	Homozygous	Based on clinical presentation; NBS was not performed
4	Metabolic crises; cyclic vomiting; epigastric pain, nausea	Female	15 years	5 years	First cousins	*ETFDH*:c.807C > T, p.Gln269His; missense	Homozygous	As part of Family screening; NBS was not performed
5	Mild glutaric aciduria type II	Female	8 years	5 months	First cousins	*ETFDH*:c.807C > T, p.Gln269His; missense	Homozygous	As part of Family screening; NBS was not performed
6	Asymptomatic	Male	10 years	2 years	First cousins	*ETFDH*:c.807C > T, p.Gln269His; missense	Homozygous	As part of Family screening; NBS was not performed
7	Vomiting; recurrent viral illness; anemia	Female	7 years	8 days	First cousins	*ETFDH*:c.807C > T, p.Gln269His; missense	Homozygous	During NBS confirmatory workup
8	Mild glutaric aciduria type 2; Repaired double aortic arch	Male	5 months	5 months	First cousins	*ETFDH*:c.807C > T, p.Gln269His; missense	Homozygous	During NBS confirmatory workup
9	Asymptomatic GA-II; Respiratory problems	Female	2 weeks	2 weeks	Not related	*ETFDH*:c.807C > T, p.Gln269His; missense	Homozygous	During NBS confirmatory workup
10	Myopathy; Hypoglycemia; Recurrent vomiting; Nausea; Hepatitis	Male	30 years	30 years	NA	*ETFDH*:c.807C > T, p.Gln269His; missense	Homozygous	Based on clinical presentation; NBS was not performed
11	Encephalopathy; vomiting; hypoglycemia; metabolic acidosis	Female	3 months	4 months	NA	*ETFDH*:c.1325C > T p.Ser442Leu; missense	Homozygous	Based on clinical presentation; NBS was not performed
12	Metabolic crises; hypoglycemia; sepsis; hyperammonemia	Female	2 days	2 months	NA	*ETFDH*:c.1414G > A p.Gly472Arg; missense *ETFB*:c.278dup p.Pro94Thrfs*; Frameshift	Homozygous Heterozygous	Based on clinical presentation; NBS was not performed
13	Recurrent vomiting; Hypoglycemia; Seizures	Female	4 years	7 years	Not related	*ETFB*:c.1035del p.Ile346Phefs*19; frameshift	Homozygous	Based on clinical presentation; NBS was not performed

NA, not available.

**Table 2:** Biochemical findings of studied patients.

Case I.D		1	2	3	4	5	6	7	8	9	10	11	12	13
**Acylcarnitine profile**	Acetylcarnitine, C2	NA	4.03 RR = 2–17.83	np	np	**31.9, H** RR = 2–27.57	np	11.14 RR = 2–27.57	15.51 RR = 2.14–15.89	5.5 RR = 2–27.57	5.5 RR = 2.–17.8	np	1.46 RR = 2–15.89	2.18 RR = 2–27.57
	Iso-/Butyrylcarnitine, C4	NA	0.18 RR < 0.83	np	np	0.62 RR < 1.06	np	**0.58, H** RR < 1.06	**0.64, H** RR < 0.46	0.48 RR < 1.06	0.36 RR < 0.83	np	**2.88, H** RR < 0.46	**0.78, H** RR < 1.06
	Isovaleryl-/2-Methylbutyrylcarn, C5	NA	0.07 RR < 0.51	np	np	**0.98, H** RR < 0.63	np	0.39 RR < 0.63	**0.44, H** RR < 0.38	**1.04, H** RR < 0.63	0.14 RR < 0.51	np	**3.41, H** RR < 0.38	0.49 RR < 0.63
	Hexanoylcarnitine, C6	NA	0.08 RR < 0.17	np	np	**0.56, H** RR < 0.23	np	**0.35, H** RR < 0.23	**0.68, H** RR < 0.14	**0.68, H** RR < 0.23	**0.23, H** RR < 0.17	np	0.03 RR < 0.14	**0.56, H** RR < 0.23
	Octanoylcarnitine, C8	NA	0.19 RR < 0.78	np	np	**1.21, H** RR < 0.45	np	**0.72, H** RR < 0.45	**1.07, H** RR < 0.19	**2.09, H** RR < 0.45	**1.99, H** RR < 0.78	np	0.12 RR < 0.19	**3.38, H** RR < 0.45
	Decenoylcarnitine, C10:1	NA	0.11 RR < 0.27	np	np	0.27 RR < 0.46	np	0.2 RR < 0.46	**0.39, H** RR < 0.25	**0.62, H** RR < 0.46	0.39 RR < 0.47	np	0.03 RR < 0.25	**0.94, H** RR < 0.46
	Decanoylcarnitine, C10	NA	0.21 RR < 0.88	np	np	**1.46, H** RR < 0.91	np	**1.4, H** RR < 0.91	**1.93, H** RR < 0.27	**4, H** RR < 0.91	**2.79, H** RR < 0.88	np	**0.28, H** RR < 0.27	**5.77, H** RR < 0.91
	Glutarylcarnitine, C5-DC	NA	0.10 RR < 0.11	np	np	0.06 RR < 0.1	np	**0.12, H** RR < 0.1	**0.22, H** RR < 0.06	0.1 RR < 0.1	**0.13, H** RR < 0.11	np	**0.47, H** RR < 0.06	**0.14, H** RR < 0.1
	Dodecanoylcarnitine, C12	NA	0.08 RR < 0.26	np	np	0.35 RR < 0.35	np	**1.17, H** RR < 0.35	**2.21, H** RR < 0.18	**0.83, H** RR < 0.35	**0.58, H** RR < 0.26	np	**0.56, H** RR < 0.18	**2.12, H** RR < 0.35
	Tetradecadienoylcarnitine, C14:2	NA	0.03 RR < 0.18	np	np	0.09 RR < 0.13	np	0.11 RR < 0.13	**0.5, H** RR < 0.09	**0.19, H** RR < 0.13	0.12 RR < 0.18	np	0.07 RR < 0.09	**0.38, H** RR < 0.13
	Tetradecenoylcarnitine, C14:1	NA	0.07 RR < 0.24	np	np	0.22 RR < 0.35	np	**0.58, H** RR < 0.35	**1.8, H** RR < 0.16	**0.45, H** RR < 0.35	**0.27, H** RR < 0.24	np	**0.48, H** RR < 0.16	**0.94, H** RR < 0.35
	Tetradecanoylcarnitine, C14	NA	0.04 RR < 0.12	np	np	**0.17, H** RR < 0.15	np	**0.61, H** RR < 0.15	**1.64, H** RR < 0.11	**0.31, H** RR < 0.15	**0.14, H** RR < 0.12	np	**0.95, H** RR < 0.11	**0.57, H** RR < 0.15
	Hexadecenoylcarnitine, C16:1	NA	0.06 RR < 0.10	np	np	0.08 RR < 0.21	np	**0.34, H** RR < 0.21	**1.43, H** RR < 0.15	0.18 RR < 0.21	0.07 RR < 0.10	np	**0.82, H** RR < 0.15	**0.53, H** RR < 0.21
	Hexadecanoylcarnitine, C16	NA	0.10 RR < 0.23	np	np	0.17 RR < 0.52	np	**0.63, H** RR < 0.52	**1.84, H** RR < 0.36	0.22 RR < 0.52	0.12 RR < 0.23	np	**2.76, H** RR < 0.36	0.4 RR < 0.52
	Octadecanoylcarnitine, C18	NA	0.05 RR < 0.14	np	np	0.1 RR < 0.12	np	0.08 RR < 0.12	**0.73, H** RR < 0.1	0.11 RR < 0.12	0.03 RR < 0.14	np	**0.58, H** RR < 0.1	**0.13, H** RR < 0.12
**Urine organic acid**		np	np	+	N	Np	N	N	**+**	np	N	+	+	+
**Carnitine profile**	Total (nmol/mL)	45 RR = 19–59	42 RR = 34–78	**81, H** RR = 43–65	20 RR = 34–77	**86, H** RR = 35–84	16 RR = 35–84	**133, H** RR = 35–84	**81, H** RR = 17–41	**52, H** RR = 17–41	74 RR = 34–78	np	18 RR = 17–41	35 RR = 28–83
	Free (nmol/mL)	24 RR = 12–46	36 RR = 25–54	44 RR = 30–50	14 RR = 22–65	42 RR = 24–63	12 RR = 24–63	53 RR = 24–63	**44, H** RR = 10–21	**38, H** RR = 10–21	**64, H** RR = 25–54	np	4 RR = 10–21	18 RR = 22–66
	Acylcarnitine (nmol/mL)	**21, H** RR = 4–15	6 RR = 5–30	7 RR = 7–14	6 RR = 4–29	**52, H** RR = 4–26	4 RR = 4–26	**95, H** RR = 4–28	**37, H** RR = 3–24	14 RR = 3–24	10 RR = 5–30	np	14 RR = 3–24	17 RR = 3–32
	AC/FC ratio	0.9 RR = 0.1–0.7	0.2 RR = 0.1–0.8	np	0.4 RR = 0.1–0.9	**1.5, H** RR = 0.1–0.8	0.3 RR = 0.1–0.8	2.5 RR = 0.1–0.8	0.8 RR = 0.1–0.8	0.4 RR = 0.1–0.8	0.2 RR = 0.1–0.8	np	**3.5, H** RR = 0.2–1.4	0.4 RR = 0.1–0.9
**Glucose levels**	Glucose (mmol/L)	4.8 RR = 3.9–6.1	2.9, L RR = 3.9–6.1	3.4, L RR = 3.9–6.1	3.9 RR = 3.9–6.1	3.6, L RR = 3.9–6.1	3.4, L RR = 3.9–6.1	5.2 RR = 3.9–6.1	5.3 RR = 3.9–6.1	**6.5, H** RR = 3.9–6.1	2.9, L RR = 3.9–6.1	np	3.4, L RR = 3.9–6.1	2.8, L RR = 3.9–6.1
**Ammonia levels**	Ammonia (µmol/L)	Np	**52, H** RR = 9–35	**56, H** RR = 9–35	**158, H** RR = 15–51	Np	30.6 RR = 16–60	**128, H** RR = 9–35		**62, H** RR = 9–28	**97, H** RR = 16–60	**80, H** RR = 9–35	**500, H** RR = 9–35	**58, H** RR = 9–35
**Ketones in urine**	Ketones	Np	Negative	**+4**	**+3**	**+1**	np	np	Negative	**+1**	Negative	np	np	**+3**

Values higher than the reference range are shown in bold face. The reference ranges mentioned in the table are age specific. H, high; L, low; N, normal; NA, not available; np, not performed; RR, reference range; +, consistent with GA-II profile.

**Table 3:** In silico analysis of the studied *ETFDH* and *ETFB* variants.

Gene	Variant	SIFT	Polyphen-2	LRT	Mutation Taster	Mutation Assessor	FATHMM	PROVEAN	MetaSVM	MetaLR	REVEL	CADD	DANN	Condel	JSD
		Score	Score	Score	Score	Score	Score	Score	Score	Score	Score	Score	Score	Score	Score
*ETFDH*	R41L	0D	0.021B	0.523D	0.999D	0.496L	0.725D	0.642D	0.780T	0.897D	0.7255D	22.5	0.992	0.554D	0.735
*ETFDH*	Q269H	0D	0.905D	0.843D	0.999D	0.987H	0.834D	0.806D	0.969D	0.944D	0.834D	20.3	0.956	0.705D	0.819
*ETFDH*	S442L	0D	1.0D	0.845D	0.810D	0.99D	0.954D	0.873D	0.990D	0.985D	0.984D	26.1	0.992	0.732D	0.809
*ETFDH*	G472R	0D	0.999D	0.843D	0.810D	0.988H	0.986D	0.900D	0.986D	0.982D	0.986D	32	0.999	0.719D	0.780
*ETFB*	P94Tfs*8	-	-	-	B	-	-	-	-	-	-	-	-	-	-
*ETFB*	I346Ffs*19	-	-	-	B	-	-	-	-	-	-	-	-	-	-

SIFT (lower scores signify pathogenicity), Polyphen-2 (higher scores signify pathogenicity), LRT (A Likelihood ratio test based on two-sided *p*-value. LRT scores are calculated using nonsynonymous-to-synonymous-rate ratio and alignment of amino acid of 31 species at the studied codon. Scores range from 0–1, higher scores signify pathogenicity), Mutation Taster (employs different in silico approaches to predict the pathogenic charge of variant of unknown significance at the DNA and protein level. Higher scores signify pathogenicity), Mutation assessor (Predicts the amino acid substitution impact on the protein using the conservation of the substituted residue in protein homologs. Higher scores indicate pathogenicity), FATHMM (Functional analysis through hidden markov models), predicts the functional impact of coding variant. Higher scores signify pathogenesis. PROVEAN (protein variation effect), higher scores signify pathogenicity. MetaSVM (Meta-analytic support vector machine) and MetaLR (meta-analytic logistic regression) are ensemble-based prediction score based on the overall scores of 10 different in silico tools. Higher scores signify pathogenicity. REVEL (rare exome variant ensemble learner), higher scores signify pathogenicity. CADD (combined annotation-dependent depletion), scores range from 1 to 99. Higher scores signify pathogenicity, e.g., a score of 30 indicates a 0.1% top variant. DANN (deleterious annotation of variants based on deep neural network), higher scores signify pathogenicity. JSD (Jensen-Shannon divergence) indicate amino acid conservation score, higher scores signify better conservation. B, benign; D, deleterious; H, high; L, low; T, tolerated.

**Table 4:** I-mutant Suite based prediction of protein stability.

Variant	Stability	RI (0–10)	ΔΔG (Kcal/mol)
*ETFDH*:p.Arg41Leu	Decreased	4	−0.35
*ETFDH*:p.Gln269His	Decreased	8	−0.87
*ETFDH*:p.Ser442Leu	Decreased	9	−3.0
*ETFDH*:p.Gly472Arg	Decreased	2	−0.24

## Data Availability

Not applicable.

## Supplementary Materials

The following are available online at https://www.mdpi.com/article/10.3390/genes12091334/s1, Figure S1. Conservation of amino acid predicted by ConSurf. Square box shown in red color represents the location of mutation; Figure S2. The secondary structure of ETF-QO predicted by PSIPRED. Square box shown in red color represents the location of mutation; Table S1. MutPred2 based prediction of molecular mechanisms affected by missense variants; Table S2. Effect of variants on the structure and domains of ETF-QO predicted by HOPE; Table S3. UniProt Accession number of sequences used for protein sequence alignment.
