# Supplementary material for: Clinical, Biochemical, and Genetic Heterogeneity in Glutaric Aciduria Type II Patients
Source: Genes (Basel). 2021 Aug 27;12(9):1334. doi: 10.3390/genes12091334 (PMC8466204; doi:10.3390/genes12091334)
Supplement: Supplementary file 1 [file genes-12-01334-s001.zip › genes-1341152-supplementary.pdf]

Title

# Clinical, biochemical, and genetic heterogeneity in glutaric aciduria type II patients

Amanat Ali [1], Fatmah Saeed Ali Almesmari [1], Nahid Al Dhahouri [1], Arwa Mohammad Saleh Ali [1], Mohammed Ahmed Ali Mohamed Ahmed Aldhanhani [1], Ranjit Vijayan [2], Amal Al Tenaiji [3], Aisha Al Shamsi [4], Jozef Hertecant [4] and Fatma Al Jasmi [1,4]*

[1]Department of Genetics and Genomics, College of Medicine and Health Sciences, United Arab Emirates University, PO Box 15551, Al Ain, Abu Dhabi, United Arab Emirates

[2]Department of Biology, College of Science, United Arab Emirates University, PO Box 15551, Al Ain, Abu Dhabi, United Arab Emirates

[3]Department of Pediatrics, Sheikh Khalifa Medical City, PO Box 51900, Abu Dhabi, United Arab Emirates

[4]Department of Pediatrics, Tawam Hospital, PO Box 15551 Al Ain, United Arab Emirates

*****Correspondence: aljasmif@uaeu.ac.ae; Tel.: +971-3-7137412; Fax: +971-3-7672022

## Supplementary Information

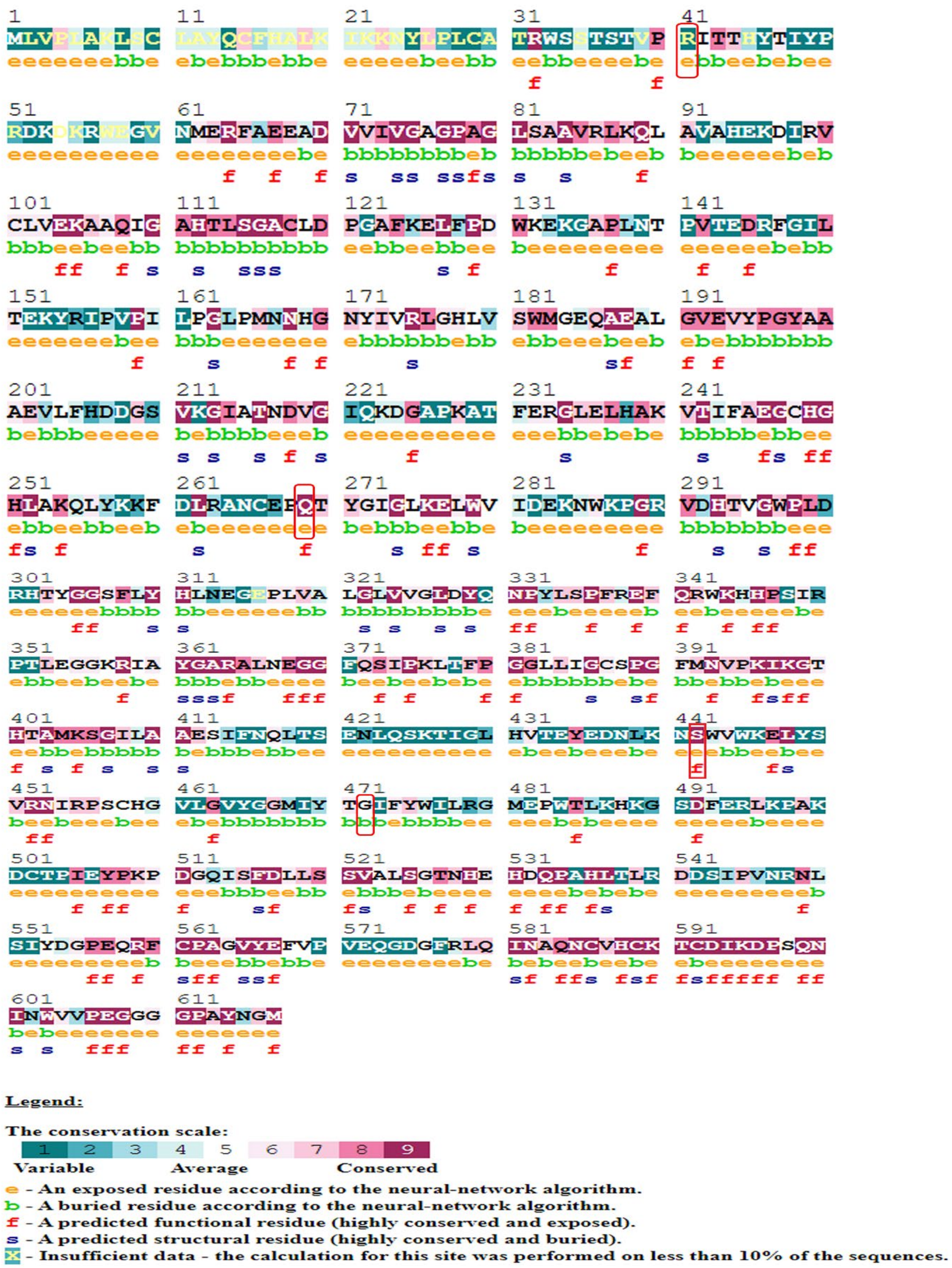


**Figure S1.** Conservation of amino acid predicted by ConSurf. Square box shown in red color represents the location of mutation.

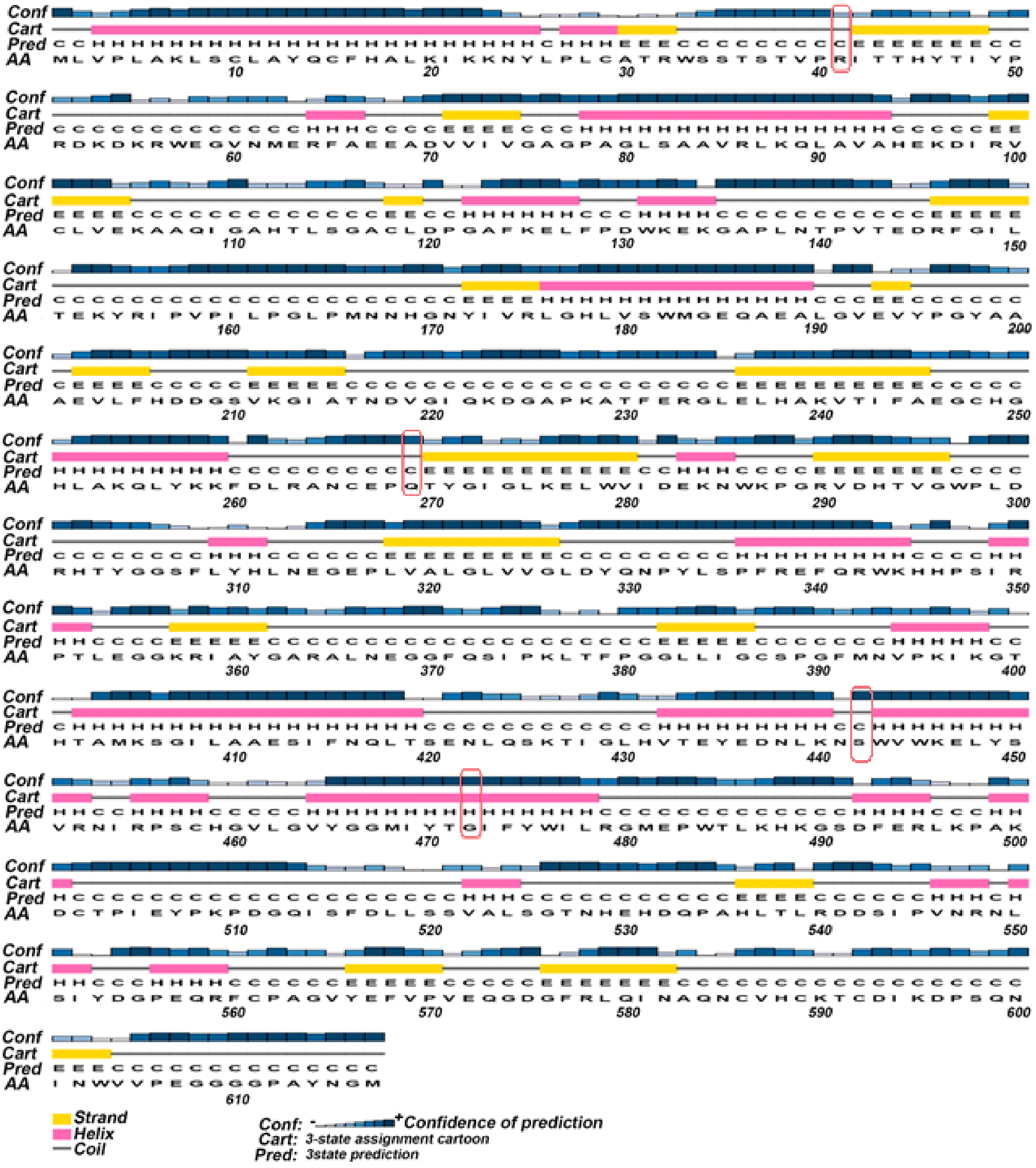


**Figure S2.** The secondary structure of ETF-QO predicted by PSIPRED. Square box shown in red color represents the location of mutation.

**Table S1.** MutPred2 based prediction of molecular mechanisms affected by missense variants.

| Variant | Molecular mechanism | MutPred2 score/g scores | *P*-value | Interpretation |
|---|---|---|---|---|
| *ETFDH*:p.Arg41Leu | Loss of glycosylation at S37 | 0.566 | 0.02 | Disease associated, actionable hypothesis |
| | Loss of disorder | 0.566 | 0.03 | Disease associated, actionable hypothesis |
| *ETFDH*:p.Gln269His | Altered Ordered interface | 0.869 | 0.02 | Disease associated, confident hypothesis |
| | Loss of Allosteric site at Y271 | 0.869 | 7.4e-03 | Disease associated, very confident hypothesis |
| | Altered Metal binding | 0.869 | 0.04 | Disease associated, confident hypothesis |
| | Loss of Catalytic site at Y271 | 0.869 | 0.01 | Disease associated, confident hypothesis |
| | Altered DNA binding | 0.869 | 0.01 | Disease associated, confident hypothesis |
| | Altered Transmembrane protein | 0.869 | 0.03 | Disease associated, confident hypothesis |
| | Loss of Relative solvent accessibility | 0.869 | 0.03 | Disease associated, confident hypothesis |
| *ETFDH*:p.Ser442Leu | Loss of ubiquitination at K440 | 0.881 | 0.04 | Disease associated, confident hypothesis |
| | Loss of disorder | 0.881 | 0.07 | Disease associated |
| *ETFDH*:p.Gly472Arg | Gain of molecular recognition features (MoRFs) binding | 0.985 | 0.004 | Disease associated, very confident hypothesis |
| | Gain of methylation at G472 | 0.985 | 0.03 | Disease associated, confident hypothesis |

Threshold $P$ value ≤ 0.05, Interpretation is performed on the basis of g and $P$ scores; actionable hypotheses: g > 0.5, $P<0.05$; confident hypotheses: g > 0.75, $P<0.05$ and very confident hypotheses: g > 0.75, $P<0.01$. Disease associated when g > 0.

**Table S2.** Effect of variants on the structure and domains of ETF-QO predicted by HOPE.

| Variant | Amino acid properties | Effect on protein structure and domain |
|---|---|---|
| *ETFDH*:p.Arg41Leu | There is a difference in charge between the wild-type and mutant amino acid. The charge of the wild-type residue will be lost, this can cause loss of interactions with other molecules or residues.<br><br>The wild-type and mutant amino acids differ in size. The mutant residue is smaller, this might lead to loss of interactions.<br><br>The mutation introduces a more hydrophobic residue at this position. This can result in loss of hydrogen bonds and/or disturb correct folding. | The mutated residue is located in a domain that is important for the main activity of the protein.<br><br>Mutation of the residue might disturb this function. |
| *ETFDH*:p.Gln269His | The wild-type and mutant amino acids differ in size.<br><br>The mutant residue is bigger, this might lead to bumps. | The mutated residue is located in a domain that is important for the main activity of the protein.<br>Mutation of the residue might disturb this function. |
| *ETFDH*:p.Ser442Leu | The wild-type and mutant amino acids differ in size.<br><br>The mutant residue is bigger, this might lead to bumps.<br><br>The mutation introduces a more hydrophobic residue at this position. This can result in loss of hydrogen bonds and/or disturb correct folding. | The residue is located in a domain that was annotated as an intramembrane region.<br><br>The wildtype residue was probably making hydrophilic interactions with other residues. The mutation could disturb these interactions and affect the structure of the intramembrane domain.<br><br>The mutant residue Leu442 will probably not fit here and bump into other residues and/or lipid molecules, thereby could disturb the structure of the intramembrane domain.<br>The mutated residue is located in a domain that is important for the main activity of the protein.<br><br>Mutation of the residue might disturb this function. |
| *ETFDH*:p.Gly472Arg | There is a difference in charge between the wild-type and mutant amino acid.<br><br>The mutation introduces a charge, this can cause repulsion of ligands or other residues with the same charge. The wild-type and mutant amino acids differ in size.<br>The mutant residue is bigger, this might lead to bumps.<br>The torsion angles for this residue are unusual. Only glycine is flexible enough to make these torsion angles, mutation into another residue will force the local backbone into an incorrect conformation and will disturb the local structure. | The wild-type residue is a glycine, the most flexible of all residues. This flexibility might be necessary for the protein's function.<br><br>Mutation of this glycine can abolish this function<br>The mutated residue is located in a domain that is important for the main activity of the protein.<br><br>Mutation of the residue might disturb this function. |

**Table S3.** UniProt Accession number of sequences used for protein sequence alignment.

| **Name of organism** | **Protein ETF-QO** |
|---|---|
| *Homo sapiens* (Human) | Q16134 |
| *Pan troglodytes* (Chimpanzee) | H2RB83 |
| *Mesocricetus auratus* (Hamster) | A0A1U7QWG6 |
| *Mus musculus* (Mouse) | Q921G7 |
| *Rattus norvegicus* (Rat) | Q6UPE1 |
| *Oryctolagus cuniculus* (Rabbit) | U3KM78 |
| *Felis catus* (Cat) | U5Q9M7 |
| *Vulpes vulpes* (Fox) | A0A3Q7SNG5 |
| *Equus caballus* (Horse) | A0A3Q2HQA1 |
| *Sus scrofa* (Pig) | P55931 |
| *Myotis lucifugus (*Bat) | G1PDD7 |
| *Capra hircus* (Goat) | A0A452FRH5 |
| *Bos taurus* (Bovine) | Q2KIG0 |
| *Loxodonta africana* (African Elephant) | G3TD93 |
| *Ailuropoda melanoleuca* (Panda) | G1L6D0 |
| *Gallus gallus* (Chicken) | F1NY29 |
| *Alligator mississippiensis* (American alligator) | A0A151PAY4 |
| *Physeter macrocephalus* (Whale) | A0A2Y9F9E2 |
| *Callorhinchus milii* (Shark) | V9KD07 |
| *Drosophila melanogaster* (Fruit fly) | Q7JWF1 |
